# SUICIDE AND COVID-19: A TEMPORAL-SPATIAL ANALYSIS IN THE STATE OF PARANÁ AND THE 15th HEALTH REGIONAL FROM 2010 TO 2020

**DOI:** 10.1192/j.eurpsy.2023.1647

**Published:** 2023-07-19

**Authors:** K. Silva, C. Bettoni, R. Santos, N. Schoeler, M. Porcu

**Affiliations:** Universidade Estadual de Maringa, Maringa, Brazil

## Abstract

**Introduction:**

Suicide occurs throughout all the life course, but in 2019 it was the fourth leading cause of death among 15-29 years old worldwide.

In Brazil, between 2010 and 2019, 112,230 deaths from suicide were recorded, with a 43% increase in the annual number of deaths from the beginning to the end of that period in all age groups. (SAÚDE, 2021).

**Objectives:**

To accomplish a space-time analysis of the rate of suicide deaths in the state of Paraná and in its 15th Health Regional, from 2010 to 2020, in the population aged 20 to 69 years, contrasting the pre-pandemic periods (2010 to 2019) and during the COVID-19 pandemic (2020).

**Methods:**

This is an ecological, observational, cross-sectional, retrospective study, using spatial and temporal analysis tools, based on secondary data obtained from the Department of Informatics of the Unified Health System (DATASUS), in the period 2010 and 2020, referring to the 5568 Brazilian cities, with emphasis on the 399 of the state of Paraná and the 30 cities of its 15th Health Regional (HR).

Exploratory Spatial Data Analysis (AEDE) techniques were used through GeoDA (version 1.20.0.8) and QGis (version 3.22.4) software to determine the existence of Spatial Autocorrelation (Moran’s Global Index or Moran I) and to calculation and visualization of the Local Spatial Association Indicator (LISA).

Global (Moran I) and local (LISA) spatial autocorrelation coefficients were considered significant when p < 0.05 at a 95% confidence level.

The Space Time Cube is a simultaneous spatial and temporal analysis methodology of ArcGIS Pro Software (ESRI, 2011) that evaluates the behavior of a value in space over a defined time interval.

**Results:**

The arithmetic mean of the age at death by suicide, from 2010 to 2020, was 41.1 years, with a standard deviation of 13.3 years and a median of 40 years. Most deaths in the pre-pandemic period occurred among men aged 20 to 29, followed by women aged 30 to 39 years and men aged 40 to 49 years.

Regarding the most common causes and modes of suicide in the State, there was no significant difference between the periods from 2010 to 2019, pre-pandemic reference, and 2020 (pandemic) in a global perspective. The three main causes of death were: 1) Hanging, strangulation or suffocation (ICD-10 X70, T71) were the majority and accounted for more than half of all causes; 2) Self-harm by gunshot (ICD- 10 X74 and X72); 3) Self-poisoning by pesticides and drugs, respectively (ICD-10 X68 and X61).

**Conclusions:**

This study showed an increase in suicide deaths in the state of Paraná, when analyzing the periods before and during the COVID-19 pandemic. The most affected population was men, aged 20 to 29 years.

**Disclosure of Interest:**

None Declared

